# Neurofeedback training improves episodic and semantic long-term memory performance

**DOI:** 10.1038/s41598-021-96726-5

**Published:** 2021-08-26

**Authors:** Yu-Hsuan Tseng, Kaori Tamura, Tsuyoshi Okamoto

**Affiliations:** 1 Graduate School of Systems Life Sciences, Kyushu University, 744 Motooka, Nishi-ku, Fukuoka Japan; 2 Faculty of Information Engineering, Fukuoka Institute of Technology, 3-30-1 Wajiro-higashi, Higashi-ku, Fukuoka Japan; 3 Faculty of Arts and Science, Kyushu University, 744 Motooka, Nishi-ku, Fukuoka Japan

**Keywords:** Learning and memory, Electroencephalography - EEG

## Abstract

Understanding and improving memory are vital to enhance human life. Theta rhythm is associated with memory consolidation and coding, but the trainability and effects on long-term memory of theta rhythm are unknown. This study investigated the ability to improve long-term memory using a neurofeedback (NFB) technique reflecting the theta/low-beta power ratio on an electroencephalogram (EEG). Our study consisted of three stages. First, the long-term memory of participants was measured. In the second stage, the participants in the NFB group received 3 days of theta/low-beta NFB training. In the third stage, the long-term memory was measured again. The NFB group had better episodic and semantic long-term memory than the control group and significant differences in brain activity between episodic and semantic memory during the recall tests were revealed. These findings suggest that it is possible to improve episodic and semantic long-term memory abilities through theta/low-beta NFB training.

## Introduction

Memory plays a key role in human life. Long-term memory is needed to retrieve necessary details for learning, working, and socializing in normal life^1^. Long-term memory is the ability to store information for a long time and infinitely and is generally classified by explicit and implicit memories. Implicit memory refers to unconscious memory, and explicit memory has been defined as information that can be consciously recalled and divided into episodic and semantic memories, the well-known types of long-term memory^2,3^. Episodic memory refers to the memory of personally experienced events, and semantic memory refers to the repository of general knowledge such as word meaning^4,5^.

The intervention of cognitive function including memory has been investigated and many training methods have been proposed. Previous studies have proposed effective methods to improve cognition and avoid certain diseases. For example, exercise interventions have been reported to reduce cognitive decline and Alzheimer’s disease^6^. Neurofeedback (NFB) is a powerful tool to improve memory ability. Real-time electroencephalogram (EEG) NFB has been shown to be effective for the self-regulation of individual brain activity and has been applied in cognition^7^, sports^8^, Alzheimer﻿’s disease^9^, and memories such as working memory^10^ and episodic memory^11^. Previous studies have reported that participants can modulate and increase the EEG band power via NFB training, resulting in improved memory^12^. In particular, episodic memory, or the retrieval of encoded information in a short time, was markedly improved after NFB training^13^. However, the effects of NFB training on long-term memory are not well-understood. Most studies only measured episodic memory in a short time (like 20 min after the encoding task)^14,15^ and did not report the forgetting rate.

Several previous studies have investigated the effect of memory training using NFB techniques. Results of those studies suggested that the gamma-band activity plays a role in managing the retrieval of episodic memories and affects episodic memory after gamma NFB training^16,17^. NFB training for the enhancement of episodic memory using the sensory-motor rhythm band, upper alpha band^11^, or alpha band acitiviy^14^ has been reported. Recent studies found that theta NFB training can effectively improve episodic memory^13^.

The activities of the theta and alpha bands are associated with episodic^18^ and semantic memory, respectively^19^; however, some studies have suggested that the theta band is related to both episodic and semantic memory^20^ and that there are interdependencies between episodic and semantic memory^21^. To investigate the effect of NFB training on episodic and semantic memory over a long time frame, the consolidation of memory was measured. Frontal midline theta bands have been associated with encoding and retrieving episodic memory^22^ and can improve^13^ and consolidate episodic memory. The frontal midline theta waves, like the hippocampal theta waves, play a role in the processing of memory^23,24^. Theta/beta NFB training has been used to treat attention conditions^25^, and previous studies suggest that the theta/low-beta NFB protocol can be used to affect the performance of episodic memory^15^. To our knowledge, there are fewer reports that the theta/low-beta NFB can affect the performance of semantic memory. It is necessary to investigate the effect of training on both episodic and semantic memory performance in the same protocol.

According to the above studies, we designed a theta/low-beta NFB protocol to train participants﻿’ episodic and semantic long-term memory and investigated the forgetting rate over 1 week. In this study, the theta/low-beta NFB protocol with auditory feedback was used to train participants and determine the effect on episodic and semantic memory. In order to understand the changes in the forgetting rate of the participants over a longer time frame after participants encoded content on the first day, we extended the test time for detecting memory ability to 1 week. It is worth investigating whether NFB training can efficiently improve the ability of episodic and semantic long-term memory or not.

We hypothesized that the theta/low-beta neurofeedback training would affect brain activities and performance of episodic and semantic memory over a long-term period. In this study, our aims were to (1) investigate the effect of theta/low-beta neurofeedback training on episodic and semantic long-term memory and (2) to examine the change in brain activities during episodic and semantic memory tasks after neurofeedback training.

## Results

The whole experiment was divided into three stages over 3 weeks. The memory tasks were conducted in the first week and the third week, namely, the first stage and third stage, respectively. In the first stage, all individuals participated in the encoding tasks for the two types of memory, i.e. episodic and semantic memory, on the first day (Day 1), and the recall test was performed again 20 min after the encoding tasks and on the second (Day 2) and seventh days (Day 3). In the second stage, only the neurofeedback group participated, and the participants received 3 days of neurofeedback training within 1 week. Then, all individuals participated in the memory tasks with different content again in the third stage, the method was the same as the first stage.

### Ability of NFB to improve episodic and semantic memory

**Figure 1:**
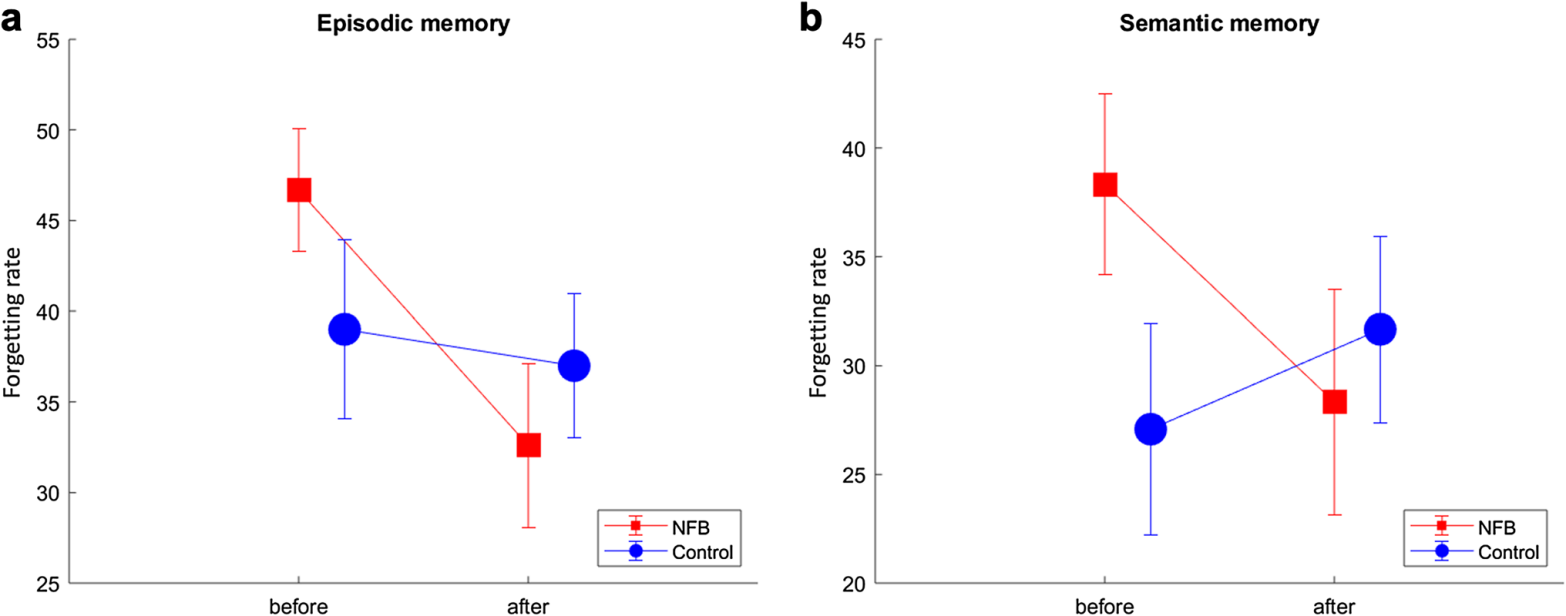
Forgetting rate for episodic and semantic memories. (**a**) The forgetting rates for episodic memory during the first (before) and third (after) stages are shown for both groups. (**b**) The forgetting rates for semantic memory during the first (before) and third (after) stages are shown for both groups. The error bars represent ± 1 standard error of the mean. This figure was drawn using MATLAB R2018a.

We calculated the forgetting rate in participants for episodic and semantic memory to examine how many items participants still remembered after a week of encoding. The forgetting rate was calculated as the correct rates on Day 1 encoded minus the correct rate on Day 3 after the encoding, both in the first and third stage. Prior to training, the episodic (t(25) = 1.3, *p* = 0.22) and semantic (t(25) = 1.3, *p* = 0.20) forgetting rates were not significantly different between the NFB and control groups. The forgetting rates for episodic memory between the groups were analysed by repeated-measures analysis of variance (ANOVA; GROUP × TIME). There was a significant main effect of TIME (F(1,26) = 7.3, *p* = 0.012, ηp^2^ = 0.0031), but no significant main effect of GROUP (F(1,26) = 0.098, *p* = 0.76, ηp^2^ = 0.071). There was a marginally significant interaction effect between GROUP and TIME (F(1,26) = 4.1, *p* = 0.053, ηp^2^ = 0.027); (Fig. 1a). The forgetting rates for semantic memory were also analysed by repeated-measures ANOVA (GROUP × TIME). There was no significant main effect of TIME (TIME: F(1,26) = 0.72, *p* = 0.403, ηp^2^ = 0.0025) and GROUP (F(1,26) = 0.46, *p* = 0.505, ηp^2^ = 0.06). There was a significant interaction between GROUP and TIME F(1,26) = 5.2 (*p* = 0.0309, ηp^2^ = 0.004; (Fig. 1b).

### Effects of NFB training

**Figure 2:**
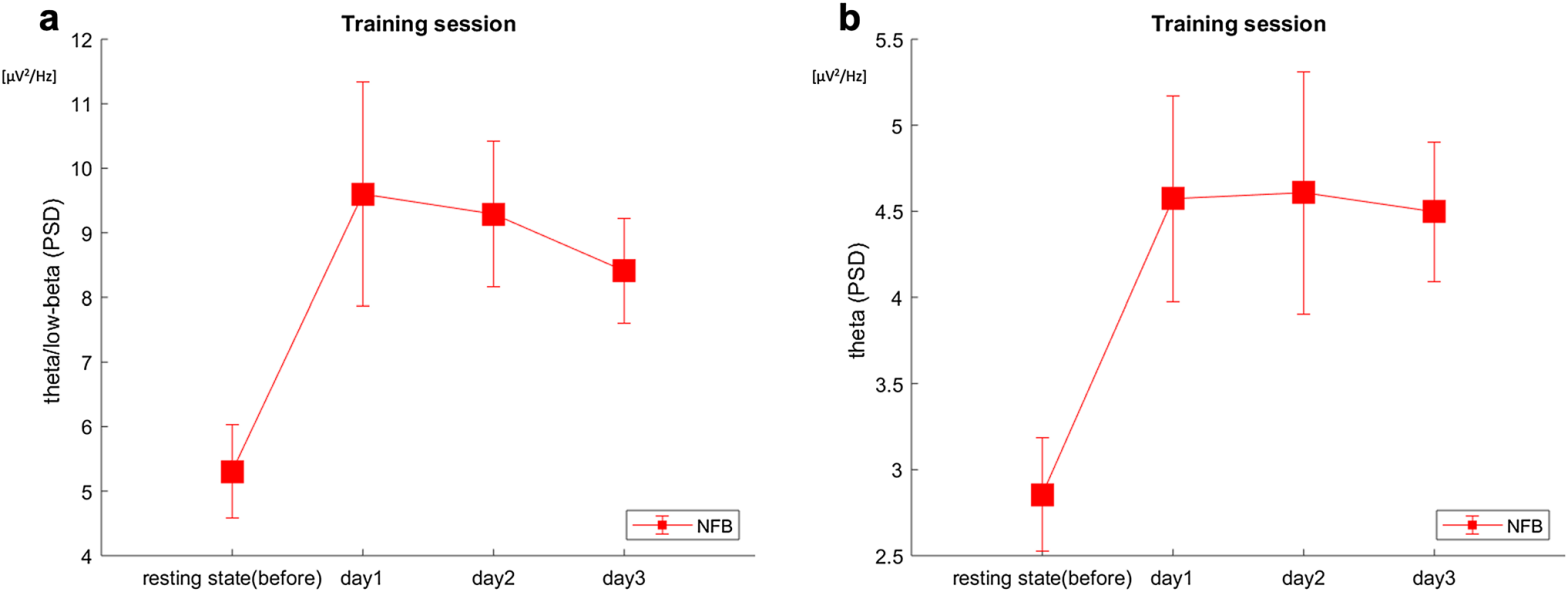
Average brain activities during neurofeedback training sessions. (**a**) The average theta/low-beta power spectral density (PSD) during the resting state and the training sessions during neurofeedback (NFB) training. (**b**) The average theta PSD during the resting state and the training sessions during NFB training. The error bars represent ± 1 standard error of the mean. This figure was drawn using MATLAB R2018a.

We confirmed the effect of training on brain activity during the 3-day NFB training. To present the within-training changes, we compared the brain activity between the first resting state, the resting state before any NFB training session on the first day, and the mean of brain activities for the six sessions for each training day. For the theta/low beta power spectral density (PSD) ratio, one-way repeated ANOVA revealed a significant main effect of the training days (F(3,42) = 4.65, *p* = 0.0068). The multiple comparison using Tukey’s HSD test showed that there were significant differences between resting and Day 1 (*p* = 0.0096), Day 2 (*p* = 0.018), and Day 3 (*p* = 0.079). However, there was no significant difference between Day 1 and Day 2 (*p* = 1.0), Day 2 and Day 3 (*p* = 0.92), and Day 1 and Day 3 (*p* = 0.83) (Fig. 2a). In the theta power spectral density (PSD), one-way repeated ANOVA revealed a significant main effect of the training days (F(3,42) = 7.04, *p* = 0.0006). The multiple comparison using Tukey HSD test showed that there were significant differences between resting and Day 1 (*p* = 0.0027), Day 2 (*p* = 0.0022), and Day 3 (*p* = 0.0041), but no significant difference between Day 1 and Day 2 (*p* = 1.0), Day 2 and Day 3 (*p* = 1.0), and Day 1 and Day 3 (*p* = 1.0) (Fig. 2b). The low-beta, alpha, and gamma PSD did not significantly increase during the 3-day NFB training (low-beta: (F(3,42) = 1.53, *p* = 0.22); alpha: (F(3,42) = 1.3, *p* = 0.29); gamma: (F(3,42) = 1.67, *p* = 0.19).

### EEG characteristics of the memory task

**Figure 3:**
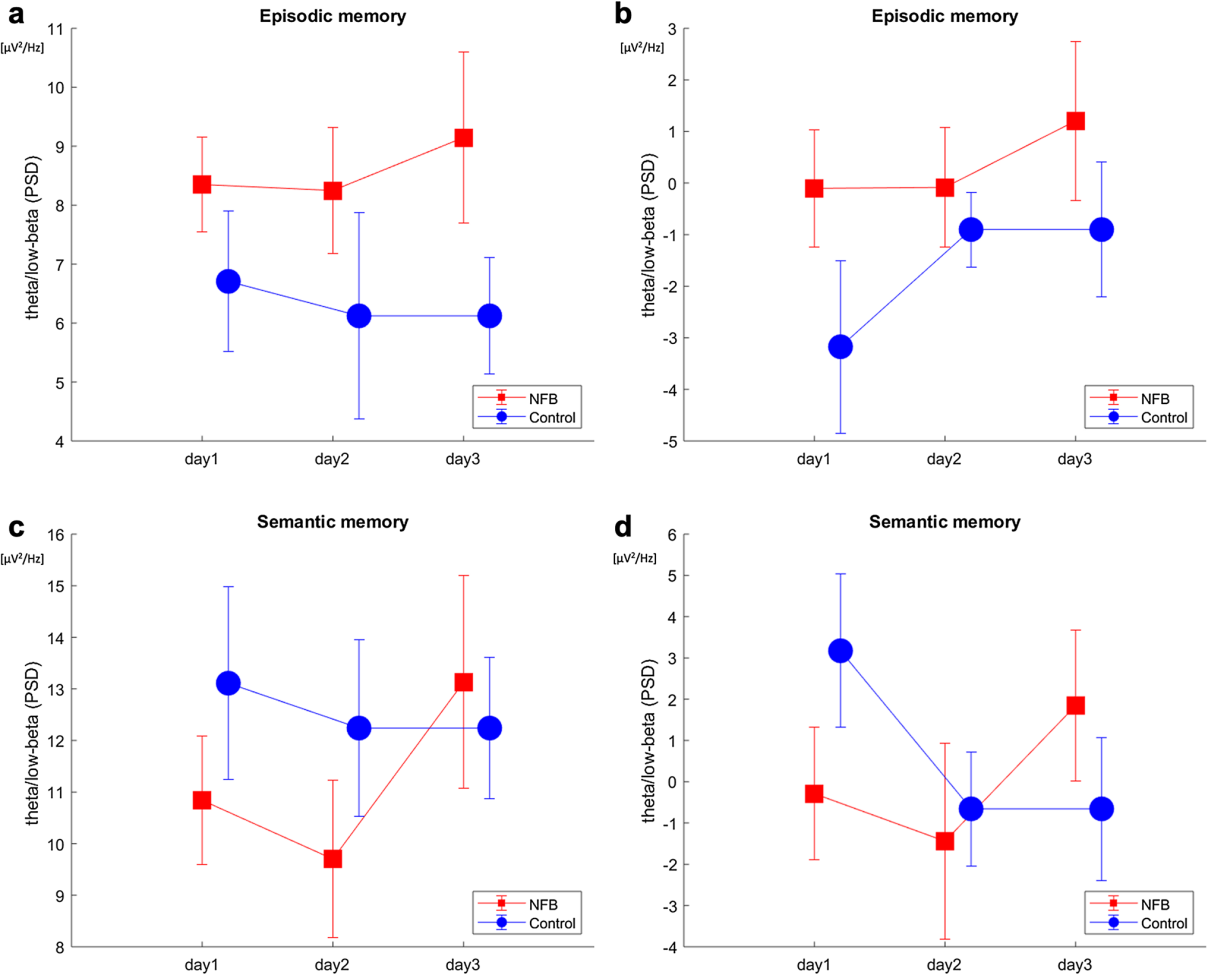
Power spectral density of theta/low-beta in the recall tests of episodic and semantic memories. (**a**): The average theta/low-beta power spectral density (PSD) during the third stage for episodic memory is shown. (**b**) : The average difference in the theta/low-beta PSD for episodic memory between the third and first stages (third stage – first stage) is shown. (**c**): The average theta/low-beta PSD in the third stage for semantic memory is shown. (**d**): The average difference in the theta/low-beta PSD for semantic memory between the third and first stages (third stage – first stage) is shown. The error bars represent ± 1 standard error of the mean. This figure was drawn using MATLAB R2018a.

The theta/low-beta PSD in all recall tests between the groups was analysed by repeated-measured ANOVA (GROUP × TIME). The results of the theta/low-beta PSD for episodic memory in the third stage (after training) show there was a marginally significant main effect of GROUP (F(1,26) = 3.7, *p* = 0.066, ηp^2^ = 0.0051), but no significant main effect of TIME (F(1,26) = 0.099, *p* = 0.75, ηp^2^ = 0.031) or interaction effect of GROUP and TIME (F(1,26) = 1.9, *p* = 0.17, ηp^2^ = 0.038) (Fig. 3a). The results of the difference in theta/low-beta PSD (third stage minus the first stage) in the recall test of episodic memory show there was a marginally significant main effect of GROUP (F(1,26) = 4.1, *p* = 0.054, ηp^2^ = 0.004), but no significant main effect of TIME (F(1,26) = 0.79, *p* = 0.38, ηp^2^ = 0.0069) or interaction effect of GROUP and TIME (F(1,26) = 0.8431, *p* = 0.36, ηp^2^ = 0.0011) (Fig. 3b). The results of the theta/low-beta PSD for semantic memory in the third stage (after training) show there was no significant main effect of GROUP (F(1,26) = 0.016, *p* = 0.9, ηp^2^ = 0.0097) or TIME (F(1,26) = 0.506, *p* = 0.48, ηp^2^ = 0.064). There was a significant interaction effect of GROUP and TIME (F(1,26) = 8.8, *p* = 0.0045, ηp^2^ = 0.0031; Fig. 3c). The results of the difference of theta/low-beta PSD (third stage minus the first stage) in the recall test of semantic memory show there was no significant main effect of GROUP (F(1,26) = 0.0121, *p* = 0.9, ηp^2^ = 0.0015) or of TIME (F(1,26) = 0.49, *p* = 0.49, ηp^2^ = 0.0022). There was a significant interaction effect of GROUP and TIME (F(1,26) = 4.3, *p* = 0.044, ηp^2^ = 0.0061; Fig. 3d). These results suggest that participants in the NFB group achieved higher theta/low-beta PSD for episodic memory after training than participants in the control group. The theta/low-beta PSD for semantic memory was increased in the NFB group after training and was higher than that in the control group on the final day.

**Figure 4:**
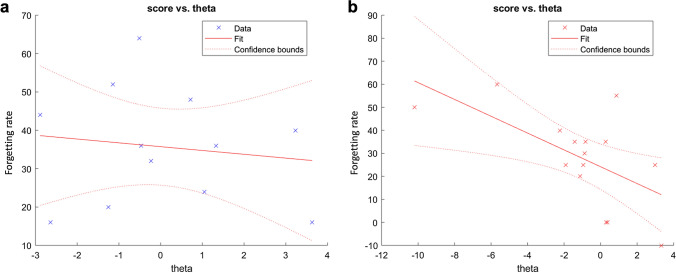
The relationship between the forgetting rate and theta power spectral density difference in semantic memory. (**a**) The relationship between the forgetting rate and the difference in theta power spectral density (PSD) for semantic memory in the control group is shown. The forgetting rate is calculated as the correct rates of the 7th day﻿’s score minus the correct rates of the 9th day﻿’s score. (**b**) The relationship between the forgetting rate and the difference in theta PSD for semantic memory in the NFB group is shown. The dotted line represents the confidence bounds. This figure was drawn using MATLAB R2018a.

The forgetting rate for episodic memory after training was not significantly correlated with the difference in theta (NFB group: b = 1.44, *p* = 0.1748; Control group: b =  − 0.43, *p* = 0.67) or theta/low-beta (NFB group: b = 0.13, *p* = 0.9001; Control group: b =  − 0.26, *p* = 0.804) in either group. The difference in theta and the forgetting rate for semantic memory after training were not significantly correlated in the control group (b =  − 0.43, *p* = 0.68; Fig. 4a), but these differences were significantly correlated in the NFB group (b =  − 2.7, *p* = 0.018; Fig. 4b). The difference in theta/low-beta was not significantly correlated in either group (NFB group: b = 0.13, *p* = 0.9, Control group: b = 0.27, *p* = 0.079). These results indicate that the forgetting rate for semantic memory was inversely proportional to the theta PSD difference after NFB training.

### Results for working memory

The correct rates of working memory between the groups were analysed by repeated-measured ANOVA (GROUP × TIME). There was no significant main effect of GROUP (F(1,26) = 0.6, *p* = 0.78, ηp^2^ = 0.0041), TIME (F(1,26) = 7.3, *p* = 0.61, ηp^2^ = 0.023), or interaction effect between TIME and GROUP (F(1,26) = 0.08, *p* = 0.9, ηp^2^ = 0.0074).

## Discussion

In the present study, we investigated whether theta/low-beta neurofeedback training would affect brain activity and performance of episodic and semantic memory over a long-term period. We showed that theta/low-beta NFB training improved episodic and semantic long-term memory. The theta/low-beta PSD of participants in the NFB group significantly increased during training sessions. The participants in the NFB group showed better performance than those in the control group after training, and this effect lasted for 1 week. The brain activities of the NFB and control groups were different for episodic and semantic memory in the third stage, after neurofeedback training. The NFB group showed higher theta/low-beta than the control group during the three recall tests for episodic memory in the third stage. In the semantic memory task, the NFB group showed higher theta/low-beta than the control group after only 1 week of encoding in the third stage. The difference in brain activity might imply that the effect of NFB training on semantic memory would appear after 1 week. These results suggest that theta/low-beta NFB training is effective to improve episodic and semantic long-term memory.

In order to achieve our purpose of studying long-term memory, we designed the memory tasks for as long a period as we could, i.e. 1 week. We performed three recall tests in a week to grasp the memory content that the participants can keep in a week. In the episodic memory task, the participants needed to remember as many pictures as they could from one hundred pictures with only one viewing opportunity. In the semantic memory task, the participants needed to remember forty monsters and the IDs of monsters by watching them five times. The memory load on these two types of memory tasks is quite large. Although the results show that after a long time scale such as 1 week, the content of the participants’ memory gradually declines with the passage of time, some of the content can still be accurately recalled in the recall test after a week (e.g. they can accurately provide some IDs of the monster). These results, such as the decay of memory content over time and the ability to remember complex content for a long time, prove that our design can achieve our goal of understanding the changes in the participant’s episodic and semantic long-term memory ability.

We designed the theta/low-beta NFB protocol and introduced auditory stimuli based on previous studies that used visual stimuli^15^. Additionally, other studies suggested that theta waves may be involved in the consolidation of episodic memory^15,26^, which we included in our training design. We considered that auditory feedback results in less disturbance than visual stimulation and allows participants to concentrate on appropriately regulating their cognitive control. During the NFB training sessions, the participants followed our instructions and regulated their cognitive process using real-time auditory feedback to improve the theta/low-beta PSD as much as possible. According to our results, the theta/low-beta PSD of participants in the NFB group increased, and the theta PSD was significantly increased during the 3-day training sessions. The increments of theta/low-beta PSD and theta PSD did not induce low-beta, alpha, or gamma PSDs during the training sessions. These findings are consistent with those of a previous study that suggested the specificity of target frequency bands^27^. Theta/low-beta protocols can effectively improve episodic memory, but low-beta/theta NFB protocols do not show the same trend^15^. The results of the theta/low-beta PSD and theta PSD did not increase linearly over the course of the second stage, similar to previously-reported findings that brain activities increase in an unstable manner during training^13,14,28–30^. We assumed that synaptic consolidation is likely to occur in NFB training, and gaps of several days during training sessions affect neuronal changes^31^. Despite different timelines, the results of this study are consistent with those of previous studies, suggesting that cognitive control processes are related to theta oscillations^32,33^ and that the theta oscillations can be trained using theta/low-beta NFB^15^. Taken together, these results indicate that the theta/low-beta NFB training protocol designed for use in this study to increase theta PSD is effective.

According to our results, the forgetting rate of participants in the NFB group significantly decreased for semantic memory and showed a marginally significant change for episodic memory after NFB training. In this study, the recall tests were conducted approximately 20 min, 1 day, and 6 days after memorization. The recall test results for episodic memory obtained 20 min after memorization were similar to previously reported results^14,15^. The NFB group performed better in the recall tests than the control group at all time points. Although our training protocol required only 3 days to train the participants, the effects of NFB training remained for 1 week.

Previous studies have indicated that theta oscillations play an important role in the memory process^24,34^. Some studies have suggested that the hippocampus theta^18^ and the increment of the theta band power in the posterior hippocampus^35^ can be used to predict successful encoding in episodic memory. In this study, our experimental device could only record the brain activities from the surface of the brain; therefore, we could not identify differences in brain activities between the different groups during the encoding task for episodic memory. However, several previous studies have suggested that the increment of theta activity affects episodic retrieval^34,36,37^. In our study, the theta activities in the NFB group were higher than in the control group during the recall test in the third stage (after NFB training sessions). These results are consistent with those of previous studies^15^ and suggest that theta/low-beta NFB training affects subsequent recall tasks of episodic memory for at least 1 week.

Participants in the NFB group had improved performance for semantic memory after theta/low-beta NFB training. In this study, regression analyses revealed that the forgetting rate for semantic memory and the difference in theta PSD between 7th and 9th days after NFB training were negatively correlated. These differences were not correlated in the control group. This finding suggested that individual theta power, which is related to the sematic processes, was significantly changed after NFB training sessions in the NFB group. This study confirms that theta power is associated with encoding and retrieval in semantic memory. Retrieval of semantic information is commonly seen in language processing. Several studies have shown that theta power supports the process of semantic memory, and that theta power increases during the retrieval of semantic information^38–42^.

To our knowledge, there are no previous reports of the use of NFB training to improve semantic memory, though several studies have reported improvement in episodic memory after NFB training^13–15^. In this study, we found that both episodic and semantic memories were improved by the theta/low-beta NFB training protocol we designed.

The hippocampus and prefrontal cortex (PFC) play important roles in episodic memory processing^43,44^. Previous EEG source estimation studies have suggested that frontal midline theta waves are generated by the anterior cingulate and medial PFC^45–48^. In addition, other reports have suggested that theta oscillations generated from the PFC in patients with epilepsy can predict the success of coding in episodic memory^49,50^. Individuals with stronger hippocampus-PFC connectivity can encode pictures into long-term memory better during working memory tasks, suggesting that the relationship between theta activity and memory encoding may depend on the connectivity between the hippocampus and PFC^51^. In this study, the theta PSD and memory scores were improved after NFB training, indicating that the training protocol used in this study may also affect the deep neural mechanisms including the hippocampus and PFC.

The theory of synaptic and systems consolidation includes an important brain circuit that is necessary for transforming short-term memory into longer-term memory^31^. The transition from short-term to long-term memory is not instant and requires repeated consolidation and other factors (including sleep)^31,52–54^. In this study, we assumed that the mechanism of NFB training sessions included the process of synaptic consolidation. The study design (six sessions of NFB training per day for 3 days) allows for the enhancement of theta, leading to synaptic consolidation and the translation of the consolidated systems. Therefore, NFB training may not lead to short-term results but lead to long-term consolidation.

The results of the recall test for semantic memory were significantly different compared to those for episodic memory. Several studies have reported that theta/beta NFB protocols modulate the medial frontal areas, which are involved in some cognitive processes including the process of response inhibition^25^. In addition, the oscillating inhibition model explains the relationship between theta oscillation and retrieval processes that induce forgetting and proposes that theta oscillation inhibits competitive memory^55–57^. The theory of the process of response inhibition and the oscillating inhibition model may explain why the participants in the NFB group achieved better results for the recall test for episodic memory and had higher theta/low-beta amplitudes. The results of semantic memory in the recall test showed a difference with episodic memory, indicating that the mechanisms of these two types of memory are different. The oscillating inhibition model explains why participants in the NFB group had higher theta/low-beta PSD for semantic memory than those in the control group on the final day of the memory period. As test day lag increases, the theta/low-beta PSD increases to inhibit the interference with competing retrieval from target memories as task difficulty increases. The theory of synaptic and systems consolidation proposes that the influence of NFB training on deep neural mechanisms will not be immediately apparent.

Although episodic and semantic memories are different in terms of the memory system or memory recall mode, some interaction between these two types of memory during the memory process via theta oscillations has been reported^20,58^. In this study, we did not find evidence of a correlation between episodic and semantic memory in the participants’ performances or brain activities. Future studies should focus on understanding how episodic and semantic memory interact during memory processing. A new method to differentiate the two types of memory is necessary to understand their independence and interaction.

In this study, participants in the NFB group received only 3 days of NFB training within 1 week. We believe that longer training periods would affect long-term memory more significantly, so we plan to design a longer-term period for the training session in our future works. It was difficult to encourage the participants to return after a 1-month gap between memory tasks. Therefore, this study was designed to include a maximum of 1 week between the recall test and the encoding task for the memory stage. We also plan to design a longer time period experiment for the recall test and encourage participants to come back again after 1 month, 6 months, and even 1 year. The sample size was determined by past studies which investigated the effect of neurofeedback training on the memories^13,14,59^. In these studies, the number of participants per group was 10 to 25. However, the effect of neurofeedback training should be examined with a larger sample size. We did not include a sham NFB control group in this experiment. Although past studies showed that the sham NFB group did not have improvements in memory performance^15^, it is necessary to assess the effect in a sham-feedback group in future work.

This study explored the trainability of long-term memory and found that NFB training can effectively improve episodic and semantic memory. In addition, we found that the brain activities after NFB training are different in the retrieval task for two different types of long-term memory. These results may provide the cornerstone for understanding long-term memory and might be used to treat memory disorders such as in patients with epilepsy and theta dysfunction or Alzheimer﻿’s disease.

## Methods

### Participants

Thirty-two students were recruited from Kyushu University (9 females, 23 males; all right-handed; age range = 18–35 years; mean age = 21.6 years, SD = 4.15 years) and randomly assigned into the NFB (n = 17) and control (n = 15) groups. All participants had normal hearing in both ears at 60 dB for all frequencies. Participants with a history of neuropsychiatric illness, hearing impairment, or a prior experience with NFB training were excluded from the study. All participants provided written informed consent for their participation in this study. The study was approved by the local ethics committee of the Faculty of Arts and Science, Kyushu University and was conducted in accordance with relevant guidelines and regulations. After excluding five participants’ data from the analyses due to recording errors, the final analysis included 15 participants in the NFB group and 12 participants in the control group.

### Procedure

**Figure 5:**
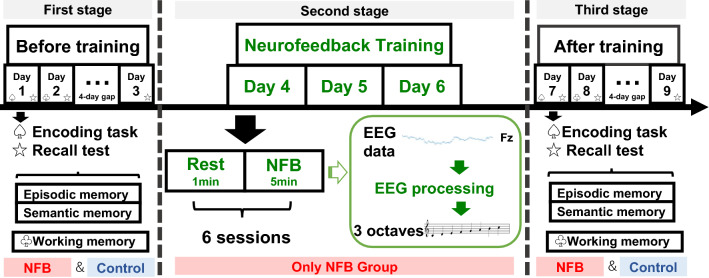
Study procedure. The study included three stages: before training, neurofeedback (NFB) training, and after training. The NFB group performed all three stages, and the control group performed the first and third stages. The first and third stages followed the same design, though the tasks differed. On days 1 and 7, participants performed the encoding tasks for both episodic and semantic memory, then performed the recall tests for both episodic and semantic memory after a 20-min wait. On days 2 and 8, participants performed the recall test for both episodic and semantic memory, then they performed the working memory task. On days 3 and 9, the participants performed another recall test for both episodic and semantic memory. This third recall test was completed after a 4-day gap. In the second stage, participants in the NFB group underwent 3 days of NFB training. One session consisted of a 1-min rest period and 5 min of auditory NFB training. There were six sessions on each training day. This figure was drawn using Microsoft PowerPoint for Mac (version 16.50).

In the NFB group, three stages were conducted over a 9-day study period (Fig. 5). In the first stage, participants were asked to perform the first set of memory tasks (see “Memory Tasks” below). This stage was completed on the first, second, and seventh days of week one. In the second stage, the participants received 3 days of theta/low beta NFB training. The 3 days of training were scheduled within 1 week (week two), as convenient for each participant. Each 30-min training session was divided into six parts (see “NFB training tasks” below). In the third stage, the participants were asked to perform the second set of memory tasks. This stage was completed on the first, second, and seventh days of week three.

In the control group, the participants completed the first and third stages only; they did not participate in the second stage and did not receive NFB training. The sets of memory tasks completed during the first and third stages were different. No participants knew the purpose of this experiment, and the participants were randomly assigned to two groups. The participants assigned to NFB did not know the goal of NFB training and just followed the instructions to increase the pitch of the sound as much as possible during the NFB training sessions. The participants did not show different motivations to complete the test in the two different time memory tasks. We followed the "CRED-nf best practices checklist 2020" to ensure the validity of this neurofeedback experiment (see Supplementary material)^60^.

### Memory tasks

#### Episodic memory task

**Figure 6:**
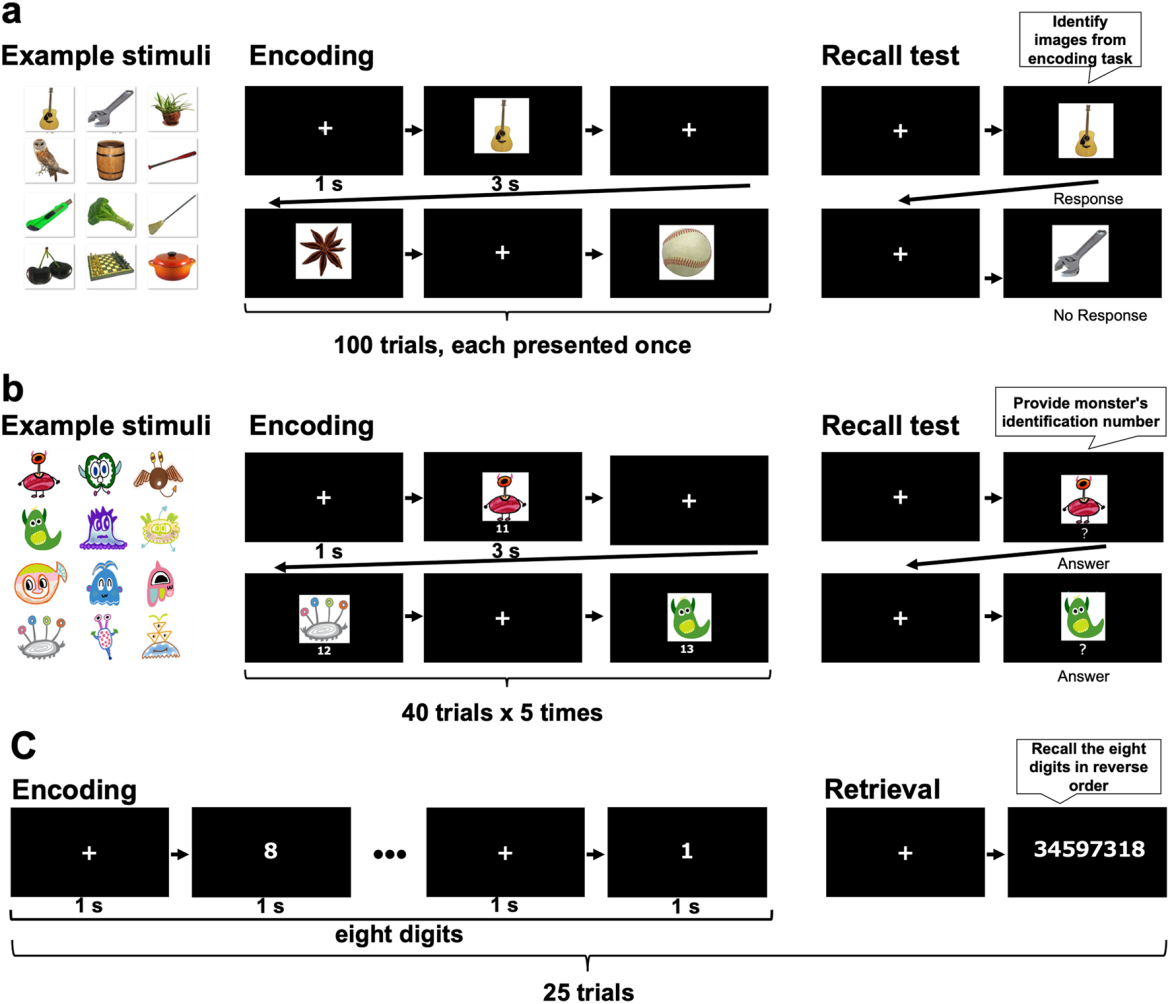
Examples of the memory tasks. (**a**) Episodic memory: In the encoding task, the participants were presented with 100 images. The recall test included 25 images from the encoding task and 75 new images. The participants were asked to identify the 25 images from the encoding task. All pictures were obtained from Mathieu Brodeur^61,62,64^ licensed under CC BY-SA 3.0 (https://creativecommons.org/licenses/by-sa/3.0/). (**b**) Semantic memory: Participants were presented with 40 images of monsters each paired with a two-digit identification number. The monster and corresponding identification number were presented five times during the encoding task. The recall test included ten monsters from the encoding task. Participants were asked to provide the identification numbers of the monsters in the recall test. All illustrations were drawn using Tayasui Sketches (https://tayasui.com). (**c**) Working memory: In each trial of the encoding task, participants were presented with eight digits. Each digit was presented for 1 s. In the recall test, participants were asked to recall the eight digits in reverse order. This task included 25 trials. This figure was drawn using Microsoft PowerPoint for Mac (version 16.50).

The episodic memory task included an encoding task and a recall test (Fig. 6a). In the encoding task, 100 images of objects (for example, various types of food, tools, and animals) selected from the normative photo dataset (The Bank of Standardized Stimuli^61,62^) were each presented once per participant. A cross mark was shown for 1 s as a fixation point before the image was presented for 3 s. One fixation/image pair was considered one trial, and 100 trials were performed. Participants were instructed to memorize the images as well as possible. Then, the participants completed old-new judgment regarding the presented images in the recall test. The recall test included 25 images that were presented in the encoding task and 75 new images. Participants used a keyboard (Numeric Keyboard for Mac, BUFFALO, Japan) to indicate when they recognized an image as one that was presented during the encoding task. The recall test was repeated three times: approximately 20 min after the encoding task and 1 and 6 days after memorization. No image was repeated between the three recall tests. The percentage of correct answers was the memory score in the recall tasks.

#### Semantic memory task

The semantic memory task followed a similar design as the episodic memory task except for the image stimuli (Fig. 6b). In the encoding task, we drew 40 monster illustrations with a two-digit identification number, which were each presented five times per participant. Participants were asked to memorize the 40 monsters and their IDs. During the recall test, 10 monsters from the encoding task were presented to the participants. Participants were asked to recall the ID number associated with each monster and enter it using a keyboard. The recall tests were conducted 20 min, 1 day, and 6 days after the encoding task. No monster image was repeated between the three recall tests. The correct rate was the memory score in the recall tasks.

#### Working memory task

We used a backward digit span task to measure the storage capacity of participants﻿’ working memory including their ability to hold and manipulate information^63^. The task included 25 trials and each trial consisted of eight digits (Fig. 6c). An image with a cross mark was presented for 1 s then the eight digits were presented for 1 s. After viewing the eight digits, the participants were asked to repeat the numbers in reverse order. One point was given for each correct digit for a maximum score of 200 points.

To understand the improvement of participants’ long-term memory ability, we calculate the forgetting rate by subtracting the last day correct rate from the first day correct rate for the first and third stages separately (first stage: day 1 correct rate − day 3 correct rate; third stage: day 7 correct rate − day 9 correct rate). The greater forgetting rate in the first stage compared to during the third stage suggested that participants memorized more and performed better in memory tasks after NFB training.

### NFB training task

The participants in the NFB group received 3 days of NFB training during the second stage of the study. NFB training included six sessions per day. Each session consisted of a 1-min resting state and 5 min of NFB training. During the resting state, participants were instructed to keep their eyes closed and being at rest. The PSD of theta (4–8 Hz)/low beta (14–18 Hz) waves in the 1-min resting state were averaged and used as the reference value. We recorded the EEG data (see “EEG recording” below) from the resting state and processed it with the theta/low beta ratio (reference value). The threshold was adjusted to be 85% of the mean theta/low beta ratio as the reference value of subsequent training. The NFB training program provided real-time positive feedback using an auditory signal, and the height of the sound was determined using the following formula: ((theta/low beta − reference value)/reference value). To familiarize the participants with the sounds and possible ranges used in the NFB training, the different pitches of sound from minimum to maximum frequency were played for the participants using 22 musical scales prior to NFB training. During the NFB training, a 60 dB of sound pressure level pure-tone beep was played for 50 ms every 2 s using double-side speakers (Companion 5 multimedia speaker system, Bose Corporation, USA)^65^. The sound was designed to be not too frequently feedbacked to avoid anxiety, or not be too infrequent to cause ineffective training effects. The range of the pitch of the sound was 130.81‒523.25 Hz during NFB, and contained a total of three octaves of intervals. Participants were instructed to keep their eyes closed and to raise the pitch of the sound as much as possible using different thinking strategies. Before NFB training, we provided a few examples of mental strategies for participants. We recommended that the participants could use any mental strategy or think in their own way freely during the NFB training. We instructed the participants that the only goal of training was to increase the pitch of the sound as high as possible. The 1-min rest periods were scheduled between each training session.

### EEG recording

Brain activities were obtained using a Quick-20 dry EEG headset (Cognionics Inc, San Diego, USA) during the memory tasks, training sessions, and 1-min resting state periods with the participants’ eyes closed. Twenty active-dry electrodes were attached according to the International 10/20 system. The reference electrode was placed on the right ear lobe (A1), and the ground electrode was placed on the upper side of the forehead (Fp1 and Fp2). Additional electrodes were placed on the right ear lobe (A2) for further analyses. The EEG channels were digitized at a sampling rate of 500 Hz. Electrode impedances were maintained below 500 kΩ.

### EEG analyses

#### Online analysis

Raw EEG data were filtered using a 60 Hz notch filter. All data were transformed into frequencies using PSD in MATLAB R2018a (MathWorks, Inc., Natick, USA). The spectral amplitudes (μV^2^/Hz) of the data were calculated and divided into the theta (4–8 Hz) and low-beta (14–18 Hz) bands from the Fz. The epoch duration was 2 s and 50% epoch overlapping was used in real-time analysis. The feedback to the participant was updated every 2 s. All data affected by eye movements or other artifacts were rejected if the absolute value of data in a half epoch (1 s) exceeded 100 μV. In the online analysis, the result was provided to the participants every 2 s. If the analytic EEG contained many artifacts, the system would not send the sound signal to the participants after 2 s. If the subsequent EEG contained fewer artifacts, a new feedback sound would be played.

#### Offline analysis

The recorded signals were re-referenced using the average of A1 and A2. All data affected by eye movements or other artifacts were rejected if the absolute value of data during 500 ms exceeded 100 μV. In addition to the previous theta and beta bands, the data were also calculated and divided into alpha (8–12 Hz) and gamma (30–55 Hz) bands from the Fz.

#### Statistical analysis

Brain activity data are presented as PSD values. The average brain wave amplitudes were calculated separately between each task/training block of each session. The repeated measures ANOVA was used to analyse the results of the forgetting rate and brain activities to determine the effect of NFB training. Regression analyses were used to examine the relationship between the results of behaviour and brain activities. Dunnett﻿’s test was used to determine the positive or negative effects of NFB training. In all statistical analyses, the significance level was set to *p* < 0.05. The data are presented with error bars to represent the standard error of the mean (SEM). All analyses were performed using JMP Pro 15 (SAS Institute Inc., North Carolina, USA).

## Supplementary Information

Supplementary Information 1.

## Data Availability

The dataset analysed and code used during the current study are available from the corresponding author on reasonable request.
