# Supplementary material for: Neurofeedback training improves episodic and semantic long-term memory performance
Source: Sci Rep. 2021 Aug 26;11:17274. doi: 10.1038/s41598-021-96726-5 (PMC8390655; doi:10.1038/s41598-021-96726-5)
Supplement: Supplementary file 1 — Supplementary Information 1. [file 41598_2021_96726_MOESM1_ESM.docx]

**Supplementary**

**Mental strategies used by participants during neurofeedback training**

After each neurofeedback training session, the participants needed to write down all mental strategies they used during this period and highlight which one was the effective strategy, and then we discussed with them how to raise the pitch of sound effects. For example, we advised the participants if they found that they have not been able to raise the pitch of the sound, they could try to switch to other mental strategies immediately. Here, we report the mental strategies that the participants used most frequently during a training session. Some of the participants tried the same way as the resting state in neurofeedback training, and according to their answers and our data, they could not increase theta or theta/low-beta by listening to sound stimuli only.

| Participant ID | DAY 1-1 | DAY 1-2 | DAY 1-3 | DAY 1-4 | DAY 1-5 | DAY 1-6 | |
| --- | --- | --- | --- | --- | --- | --- | --- |
| P01 | lyrics | lyrics | lyrics/resting | lyrics/novel | novel | lyrics | |
| P02 | name | name | name/calculate | name | name | name/event | |
| P03 | event/name/calculate | name/calculate | sound/name | calculate | event/sport | calculate | |
| P04 | face | program/calculate | people | program | event | calculate/event | |
| P05 | event | event | event | event | event | event | |
| P06 | sing/calculate | sing/calculate | sing/calculate | sing/calculate | sing/calculate | sing/calculate | |
| P07 | rest | test | idol | idol | song | travel | |
| P08 | language | language | event | travel/project | instrument | language/event | |
| P09 | sport/report | travel | rest | project | calculate | tv | |
| P10 | bike | airplane | game | sound | rest | experiment | |
| P11 | sport/fly | sport/language | sport/language/game | sport/sing | calculate/sport | calculate/tv | |
| P12 | habit/calculate | travel/habit/calculate | habit/eat/book | name | travel | name | |
| P13 | calculate/name | name | name | event | game | shiritori | |
| P14 | rest | project | event | traffic | experiment | eat | |
| P15 | name/sport | calculate/event | name | name/event | job | map/name | |
|  | | | | | | |  |
| Participant | DAY 2-1 | DAY 2-2 | DAY 2-3 | DAY 2-4 | DAY 2-5 | DAY 2-6 | |
| P01 | resting | book | resting | lyrics | lyrics | lyrics | |
| P02 | name/calculate | name/event | event/calculate | event/name | event | event/calculate/name | |
| P03 | calculate/event | song/resting | song/resting | calculate | shiritori | shiritori | |
| P04 | event | program | face/name/map | test | calculate | calculate/map | |
| P05 | event | event | event | calculate | event | event | |
| P06 | sing | sing | sing | sing | sing | sing | |
| P07 | speech | sport | sport | song | song | sport | |
| P08 | event/map/song | language/project | project/sport | map/language | travel/eat | travel | |
| P09 | report | sport | rest | sport | rest | rest | |
| P10 | sport | calculate | calculate | calculate | name | calculate/word | |
| P11 | sport/calculate/language | sport/sing/test | book/map | sport/book | language | sport/eat | |
| P12 | movie | book | shiritori/song | travel/book | movie | book | |
| P13 | calculate | calculate | calculate | calculate | calculate | create | |
| P14 | schedule | language | language | travel | sport | cook/game | |
| P15 | shiritori | project | calculate | book | shiritori/future | shiritori/sound | |
|  | | | | | | |  |
| Participant | DAY 3-1 | DAY 3-2 | DAY 3-3 | DAY 3-4 | DAY 3-5 | DAY 3-6 | |
| P01 | lyrics | resting/lyrics | lyrics | lyrics | resting | lyrics/novel | |
| P02 | event/calculate/name | name | name | name | language | sport/language/name | |
| P03 | shiritori | shiritori | shiritori | name | shiritori | shiritori | |
| P04 | event | face | test | building | pet | program | |
| P05 | event | event | event | event | event | training | |
| P06 | event | event | calculate | calculate | calculate | calculate | |
| P07 | sport | sport | test | test | win the lottery | travel | |
| P08 | calculate/project | travel | map | language/instrument/project | map/report | travel | |
| P09 | rest | sport | project/sport | sport | event | event | |
| P10 | calculate | name/rest | language | think/dream | think | project | |
| P11 | tv/book | sport/tv/space | sport/job | calculate/name | sport/eat | sport/project | |
| P12 | book | game | movie/book | sing | job/book | book | |
| P13 | calculate | calculate | calculate | shiritori | shiritori | shiritori/calculate | |
| P14 | sport | drive | sing | project | army | Language | |
| P15 | name/shiritori | tv/event | shiritori/calculate/name | shiritori | friend | friend | |

Table 1. Mental strategies used by participants during neurofeedback training

The black font indicates a successful mental strategy, and the red font indicates a failed mental strategy.

**The raw score of episodic and semantic memory**

Figure 1 shows the raw scores of episodic and semantic memory in the different groups.

**
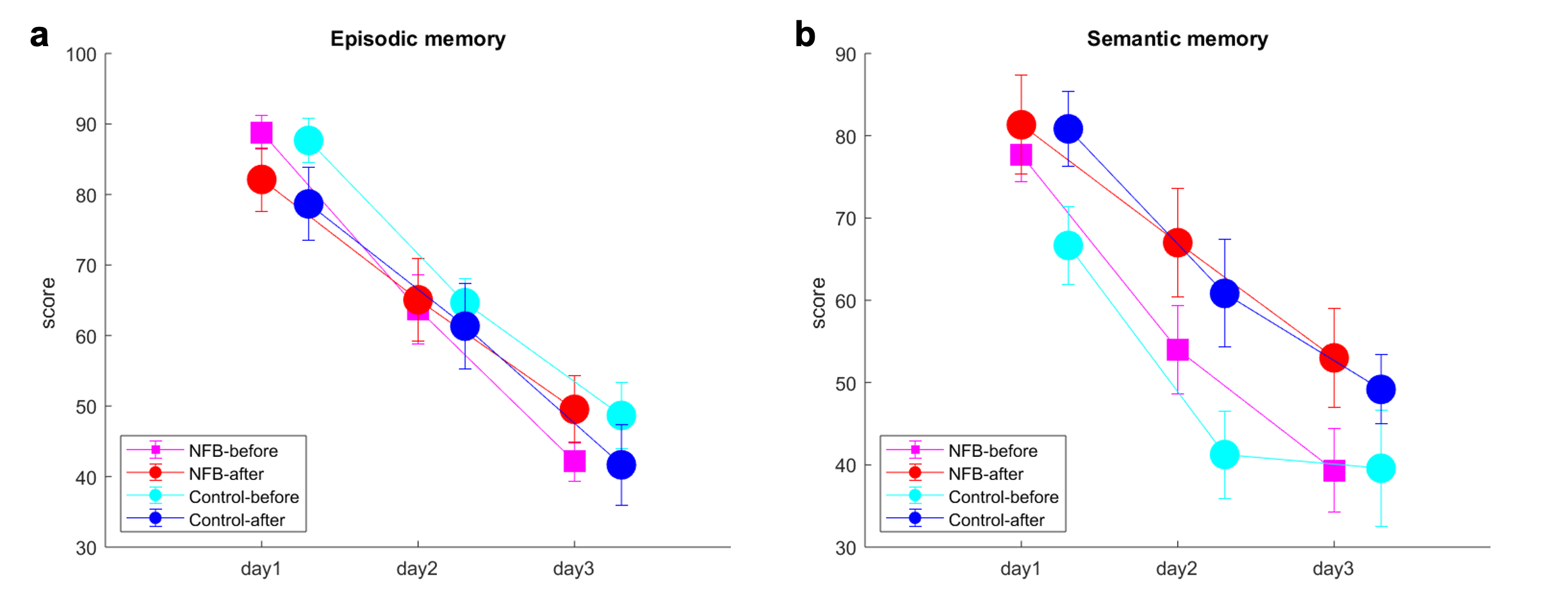
**

Figure. 1 The raw scores for episodic and semantic memory

**a**. The raw score of episodic memory. **b**. The raw score of semantic memory

**Details about the pre-experiment**

We determined that we needed about 30 participants divided into two groups as the sample size in this study. The sample size was determined according to the EEG study standard. The sample number determination corresponded to the regulation of phase one of clinical trials in Japan, which recommended the number of participants should be 10‒20 in the medical intervention experiment. We also referred to past studies investigating the effect of episodic and working memories from the neurofeedback training, which all used 10 to 25 participants per group^13,14,59^.

Before the start of the experiment, we performed preliminary experiments on seven people. First, we ensured the neurofeedback protocol we designed could provide feedback to people in real-time so that people could be effectively trained. Second, we ensured the memory tasks we designed followed the definition of episodic and semantic long-term memories, and the content of the memory was moderate. We started the formal experiment after passing the preliminary experiment.

**Checklist for the NFB experiment**

We followed the "CRED-nf best practices checklist 2020" to ensure the validity of this neurofeedback experiment.

| **CRED-nf best practices checklist 2020** | | | |
| --- | --- | --- | --- |
| **Domain** | **Item** | **Checklist item** | **Reported on page** |
| **Pre-experiment** | | | |
|  | 1a | Pre-register experimental protocol and planned analyses | See supplementary in page 2 |
|  | 1b | Justify sample size | See supplementary in page 2 |
| **Control groups** |  |  |  |
|  | 2a | Employ control group(s) or control condition(s) | See methods in page 16 |
|  | 2b | When leveraging experimental designs where a double-blind is possible, use a  double-blind | No |
|  | 2c | Blind those who rate the outcomes, and when possible, the statisticians involved | No |
|  | 2d | Examine to what extent participants and experimenters remain blinded | No |
|  | 2e | In clinical efficacy studies, employ a standard-of-care intervention group as a benchmark for improvement | No |
| **Control measures** |  |  |  |
|  | 3a | Collect data on psychosocial factors | Yes |
|  | 3b | Report whether participants were provided with a strategy | See methods in page 21 |
|  | 3c | Report the strategies participants used | See supplementary in page 1 |
|  | 3d | Report methods used for online-data processing and artefact correction | See methods in page 21 |
|  | 3e | Report condition and group effects for artefacts | No |
| **Feedback specifications** |  |  |  |
|  | 4a | Report how the online-feature extraction was defined | See methods in page 20 |
|  | 4b | Report and justify the reinforcement schedule | See results in page 5 |
|  | 4c | Report the feedback modality and content | See methods in page 20 |
|  | 4d | Collect and report all brain activity variable(s) and/or contrasts used for feedback, as displayed to experimental participants | Yes |
| **Outcome measures** |  |  |  |
| **Brain** | 5a | Report neurofeedback regulation success based on the feedback signal | See methods in page 20 |
|  | 5b | Plot within-session and between-session regulation blocks of feedback | See results in page 8 |
|  | 5c | Statistically compare the experimental condition/group to the control  condition(s)/group(s) (not only each group to baseline measures) | See results in page 9 |
| **Behaviour** | 6a | Include measures of clinical or behavioural significance, defined a priori, and describe whether they were reached | See methods in page 17 |
|  | 6b | Run correlational analyses between regulation success and behavioural  outcomes | See results in page 10 |
| **Data storage** |  |  |  |
|  | 7a | Upload all materials, analysis scripts, code, and raw data used for analyses, as well as final values, to an open access data repository, when feasible | Please contact authors |

Table 2. The checklist for the NFB experiment
